# Mild SARS-CoV-2 infection results in long-lasting microbiota instability

**DOI:** 10.1128/mbio.00889-23

**Published:** 2023-06-09

**Authors:** Vaibhav Upadhyay, Rahul K. Suryawanshi, Preston Tasoff, Maria McCavitt-Malvido, Renuka G. Kumar, Victoria Wong Murray, Cecilia Noecker, Jordan E. Bisanz, Yulin Hswen, Connie W. Y. Ha, Bharath Sreekumar, Irene P. Chen, Susan V. Lynch, Melanie Ott, Sulggi Lee, Peter J. Turnbaugh

**Affiliations:** 1 Department of Microbiology and Immunology, G.W. Hooper Research Foundation, University of California, San Francisco, California, USA; 2 Department of Medicine, University of California San Francisco, University of California, San Francisco, California, USA; 3 Department of Medicine, Benioff Center for Microbiome Medicine, University of California, San Francisco, California, USA; 4 Gladstone Institutes, San Francisco, California, USA; 5 Department of Epidemiology and Biostatistics and the Bakar Computational Health Institute, University of California San Francisco, San Francisco, California, USA; 6 Department of Pediatrics, University of California San Francisco, University of California, San Francisco, California, USA; 7 Chan Zuckerberg Biohub-San Francisco, San Francisco, California, USA; University of Maryland, School of Medicine, Baltimore, Maryland, USA

**Keywords:** COVID-19, SARS-CoV-2, non-hospitalized patients, human gut microbiome, gastrointestinal symptoms, microbial ecology

## Abstract

Viruses targeting mammalian cells can indirectly alter the gut microbiota, potentially compounding their phenotypic effects. Multiple studies have observed a disrupted gut microbiota in severe cases of severe acute respiratory syndrome coronavirus 2 (SARS-CoV-2) infection that require hospitalization. Yet, despite demographic shifts in disease severity resulting in a large and continuing burden of non-hospitalized infections, we still know very little about the impact of mild SARS-CoV-2 infection on the gut microbiota in the outpatient setting. To address this knowledge gap, we longitudinally sampled 14 SARS-CoV-2-positive subjects who remained outpatient and 4 household controls. SARS-CoV-2 cases exhibited a significantly less stable gut microbiota relative to controls. These results were confirmed and extended in the K18-humanized angiotensin-converting enzyme 2 mouse model, which is susceptible to SARS-CoV-2 infection. All of the tested SARS-CoV-2 variants significantly disrupted the mouse gut microbiota, including USA-WA1/2020 (the original variant detected in the USA), Delta, and Omicron. Surprisingly, despite the fact that the Omicron variant caused the least severe symptoms in mice, it destabilized the gut microbiota and led to a significant depletion in *Akkermansia muciniphila*. Furthermore, exposure of wild-type C57BL/6J mice to SARS-CoV-2 disrupted the gut microbiota in the absence of severe lung pathology.

**IMPORTANCE:**

Taken together, our results demonstrate that even mild cases of SARS-CoV-2 can disrupt gut microbial ecology. Our findings in non-hospitalized individuals are consistent with studies of hospitalized patients, in that reproducible shifts in gut microbial taxonomic abundance in response to SARS-CoV-2 have been difficult to identify. Instead, we report a long-lasting instability in the gut microbiota. Surprisingly, our mouse experiments revealed an impact of the Omicron variant, despite producing the least severe symptoms in genetically susceptible mice, suggesting that despite the continued evolution of SARS-CoV-2, it has retained its ability to perturb the intestinal mucosa. These results will hopefully renew efforts to study the mechanisms through which Omicron and future SARS-CoV-2 variants alter gastrointestinal physiology, while also considering the potentially broad consequences of SARS-CoV-2-induced microbiota instability for host health and disease.

## INTRODUCTION

Mammalian viruses exhibit bidirectional interactions with the gut microbiota (the trillions of microorganisms colonizing the gastrointestinal tract) (1). The gut microbiota and its aggregate gene content (the gut microbiome) contribute to protective immunity from influenza (2, 3) and respiratory syncytial virus (4), whereas human immunodeficiency virus (HIV) is associated with marked perturbations in gut microbial community structure and function (5). The gut microbiota is also distinctive in patients with severe acute respiratory syndrome coronavirus 2 (SARS-CoV-2) infections requiring hospitalization relative to healthy controls (6 - 8); however, the direct causal effects of SARS-CoV-2 relative to concomitant changes in host immunity, diet, and pharmacotherapy remain unknown. Decreased bacterial richness is a reproducible marker of SARS-CoV-2 infection (7 - 10), whereas the specific bacterial taxa correlated with infection vary between studies, in part due to differences in the built environment (10). Furthermore, the generalizability of these findings to subjects with milder cases that do not require hospitalization and have less severe symptoms is unclear.

The impact of SARS-CoV-2 in the outpatient setting is a timely question as current estimates suggest the vast majority (92%) of individuals in the USA that test positive for SARS-CoV-2 will not require hospitalization (11). The predominant variant at the time of this manuscript is Omicron (12), which is less likely to require hospitalization (13), aided in part by preexisting immunity due to vaccination and prior waves of infection (14). Despite these encouraging trends, a growing number of non-hospitalized adults still develop long-lasting symptoms persisting months after clearing the virus (15, 16), highlighting the importance of understanding the mechanisms responsible. Considered in light of emerging evidence that the microbiome can exhibit “ecological memory” of past events (17), we hypothesized that even mild cases of SARS-CoV-2 could still disrupt the gut microbiota, potentially contributing to phenotypes months later.

Here, we present an analysis of subjects participating in the COVID-19 Host Immune Response Pathogenesis (CHIRP) study. CHIRP was an exploratory study of primarily outpatients and their household contacts collected between May and August 2020. Prior work on this cohort revealed SARS-CoV-2-specific CD8^+^ T cells are maintained well into convalescence (recovery from disease), even in mild disease (18, 19). However, these prior studies did not analyze the gut microbiota. To address this knowledge gap, we used paired 16S rRNA gene and metagenomic sequencing (MGS) from longitudinal samples collected from CHIRP cases and household controls. We confirmed and extended these findings using two mouse models of SARS-CoV-2: the K18-humanized angiotensin-converting enzyme 2 (K18-hACE2) mouse model (20) and wild-type C57BL/6J mice. SARS-CoV-2 binds to the human ACE2 receptor, but some variants cannot interact with the orthologous mouse protein (21, 22). The K18-hACE2 mouse expresses human ACE2 under the keratin-18 promoter, leading to expression in lung epithelium, and provides an experimentally tractable model to study multiple SARS-CoV-2 variants (23). In contrast, C57BL/6J mice develop infection without meaningful lung pathology with a subset of variants (24), and present a complementary mouse model of infection. Taken together, our results define a significant and long-lasting impact of mild SARS-CoV-2 infection on the gut microbiota.

## RESULTS

### Lack of a reproducible shift in the gut microbiota following mild cases of SARS-CoV-2

We evaluated the gut microbiota of outpatients with SARS-CoV-2 infections during the first year of the pandemic. Samples were collected in the weeks to months after initial infection (maximum 154 days after initial positive test results; Fig. S1). Fifty-three longitudinal stool samples were collected from 18 subjects enrolled in CHIRP, including 6 men and 12 women whose ages ranged from 19 to 71 years in a Case–Control household study design (Table S1). DNA was extracted from samples and used for paired 16S rRNA gene sequencing (16S-Seq) and MGS. We generated 135,600±7,236 (mean±sem) high-quality 16S rRNA gene reads/sample and 54.0±2.19 million (mean±sem) high-quality MGS reads/sample (Table S2A).

**Fig 1 F1:**
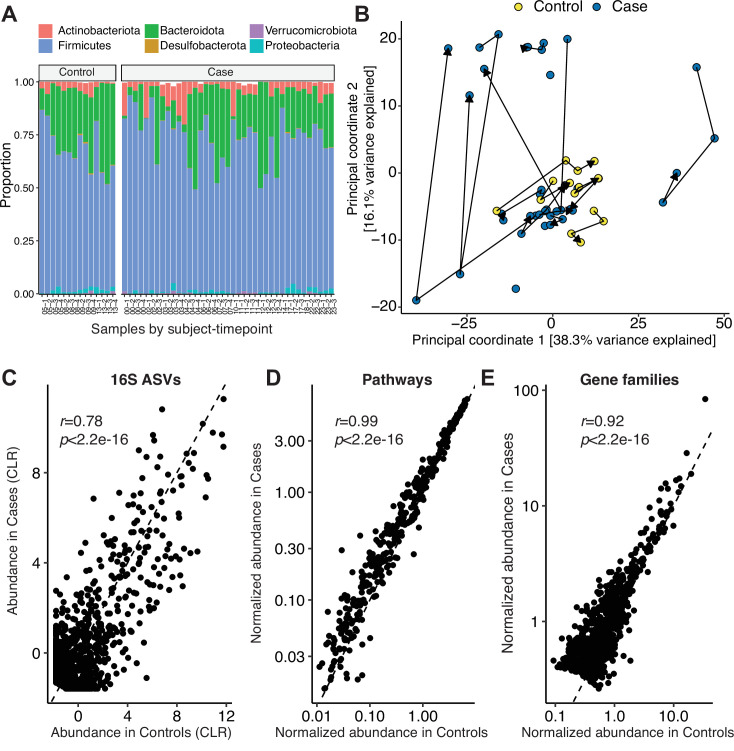
Mild severe acute respiratory syndrome coronavirus 2 (SARS-CoV-2) infection does not reproducibly change the gut microbiome months after infection. (**A**) Phylum-level taxonomic summary of 16S-Seq data for Cases and Controls. (**B**) Principal Coordinates Analysis of 16S-Seq data. Lines indicate successive samples from each individual. (**C–E**) Scatter plots reveal a significant correlation between Cases and Controls for the abundance of (**C**) 16S-Seq ASVs, (**D**) metagenomic sequencing (MGS) pathways, and (**E**) MGS gene families (*n* = 53 samples from 14 Cases and 4 Controls). Pearson’s *r* and p value annotated for (**C–E**). ASV, amplicon sequence variant; CLR, Centered Log Ratio.

On average, the gut microbiomes of SARS-CoV-2 Cases were similar to Controls. Both groups were primarily colonized by members of the *Firmicutes*, *Bacteroidota*, and *Actinobacteriota* phyla across the sampling period (16S-Seq data; Fig. 1A). PERMANOVA testing did not reveal a significant difference in gut microbial community structure when adjusted for longitudinal sampling (Case/Control *r^2^* = 0.0517 *P* = 0.00300, *P*_*adj*_ = 0.584; Days Post-PCR *r^2^* = 0.0134, *P* = 0.363, *P*_*adj*_ = 0.584; [Fig F1] Fig. 1B). Similarly, there were no changes in bacterial diversity (Fig. S2A and B) or 16S-Seq ASV abundances, which were highly correlated between groups (Fig. 1C). MGS data at the pathway (Fig. S2C) and gene family level (Fig. S2D) was not significantly different between Cases and Controls after adjustment for longitudinal sampling (Pathway Case/Control *r^2^* = 0.0356, *P* = 0.0152, *P*_*adj*_ = 0.1; Gene families Case/Control *r^2^* = 0.4611, *P* = 9.999e-5, *P*_*adj*_ = 1). Pathway (Fig. 1D) and gene families (Fig. 1E) were highly correlated between Cases and Controls. Statistical testing confirmed the lack of a reproducible shift in phylum, ASV, or metabolic pathway levels after adjustment for longitudinal sampling (*P*_*adj*_ > 0.1, see Methods section).

**Fig 2 F2:**
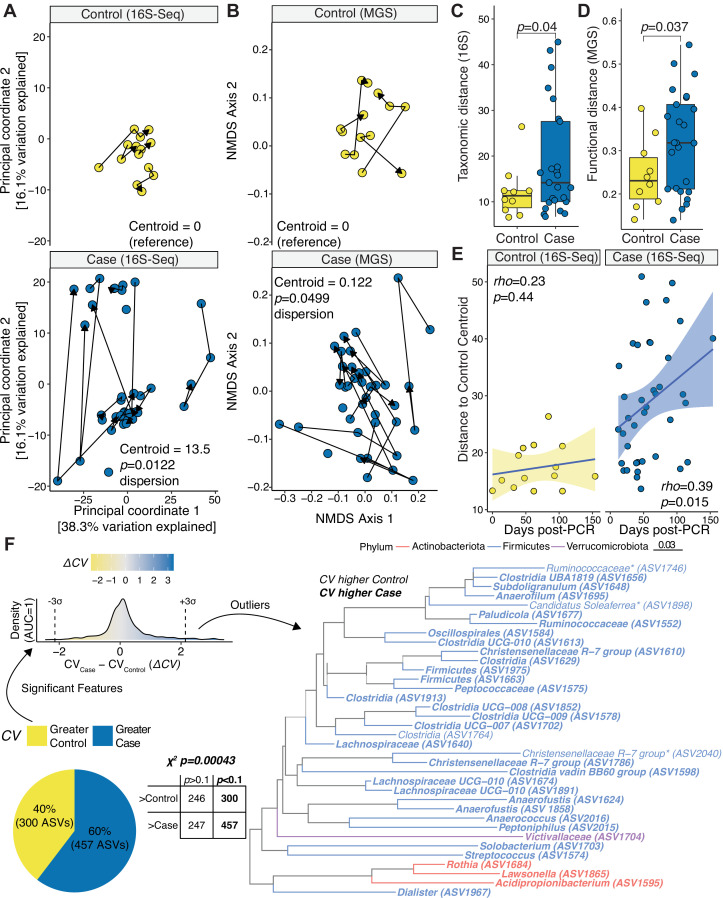
The human gut microbiota is less stable following mild severe acute respiratory syndrome coronavirus 2 (SARS-CoV-2) infection. (**A–B**) Principal Coordinates Analysis of 16S-Seq (**A**) and NMDS of metagenomic sequencing (MGS) data (**B**). Lines indicate successive samples from an individual (p, β-dispersion test with Tukey’s correction). (**C–D**) PhILR distances between successive samples for a subject by 16S-Seq (**C**) and MGS (**D**) (p values, Student’s *t* test). (**E**) Euclidean distance from each 16S-Seq sample to the Control group centroid (Spearman correlations annotated). (**F**) Comparison of amplicon sequence variant (ASV) variation. *F* statistic comparing Cases and Controls, p_adj_ < 0.1. Coefficient of variation (CV) was calculated for each ASV and group. A two-by-two table with a χ2 test annotated above the table and a pie chart depicts the proportion of significant ASVs whose CV was greater in cases or controls. Δ*CV (*CV_Case_*–*CV_Control_) for significant tests are plotted as a density curve, with three standard deviations above and below 0 annotated. A phylogenetic tree of outlier ASVs is displayed. *n* = 18 subjects, 53 samples.

### Long-lasting microbiota instability following SARS-CoV-2 infection

We hypothesized that the lack of consistent differences in the gut microbiomes of Cases and Controls may have been due to high levels of variation among the SARS-CoV-2-infected individuals. Consistent with this hypothesis, visualization of our 16S-Seq and MGS data by Principal Coordinates Analysis (PCoA) and quantification of β-dispersion (25) both demonstrated a marked and significant increase in temporal variation of the gut microbiotas of Cases relative to Controls (Fig. 2A and B). Samples from Cases deviated across sampling timepoints more than Controls in terms of 16S-Seq ASV abundance (Fig. 2C) and MGS functional pathways (Fig. 2D). Cases exhibited a significant increase in distance to Controls over time; however, there were marked fluctuations across the entire time course (Fig. 2E).

Next, we sought to identify which bacterial taxa and metabolic pathways were most variable following SARS-CoV-2 infection. We calculated the CV, a statistical measure of dispersion, for all ASVs or MGS pathways. As expected, CV was negatively correlated with ASV and pathway abundance (Fig. S3). Cases exhibited significantly higher CVs at the ASV (Fig. S4A) and pathway (Fig. S4B) level relative to Controls. We then used *F* tests to identify ASVs that had a significant difference in variability between groups: the more variable ASVs were significantly enriched in Cases (Fig. 2F, pie chart). We focused on 36 ASVs that were three standard deviations above or below an equivalent CV between groups. The majority (32/36) of these outlier ASVs were more variable in Cases than Controls. The *Firmicutes* phylum was most common, representing 32/36 outlier ASVs (Fig. 2F). We also identified three ASVs from the *Actinobacteriota* phylum, including an ASV assigned to the *Rothia* genus that has been previously associated with SARS-CoV-2 infection (9, 10).

Complementary analyses of our metagenomic data supported these overall trends (Fig. S4). Gene family abundance exhibited significantly more variability (assessed by β-dispersion) in Cases relative to Controls (Fig. S4C). Gene families trended towards more variation between sample points for Cases compared to Controls (Fig. S4D). Gene family variability assessed by CV was also significantly higher in Cases relative to Controls (Fig. S4E).

Taken together, these data indicate that the human gut microbiome can be destabilized months after initial infection with SARS-CoV-2. However, it is not possible to infer a causal role of SARS-CoV-2 infection in destabilizing the gut microbiota based only on observational studies in humans given the clear potential for confounding factors. By focusing on outpatient sampling, we were able to rule out the confounding effects of hospitalization and treatment in prior studies (26); however, our longitudinal samples corresponded to a period of extensive social distancing (Fig. S5) that could have feasibly impacted the gut microbiota (27). Thus, we turned to an established mouse model of SARS-CoV-2 infection, wherein environmental and genetic variables could be controlled to test the causal role of viral infection in shaping the mouse gut microbiota.

### SARS-CoV-2 alters the gut microbiota in susceptible mice

We tested the impact of three SARS-CoV-2 variants on the gut microbiota of K18-hACE2 mice: WA1, Delta, and Omicron (BA.1). These variants differ in their phenotypic impacts in K18-hACE2 mice, with each successive temporal SARS-CoV-2 variant resulting in milder infection than the prior variant (23). Fifty K18-hACE2 female mice were housed in an Animal Biosafety Level 3 (ABSL3) facility, and longitudinal stool samples were collected following infection and successive planned sacrifice for virological assessment of gut and lung tissues (Fig. S6A; Table S2B). At each timepoint (days 2 and 4), an entire cage of mice was sacrificed for viral titer assessment from WA1, Delta, and Omicron infected cages, and all remaining mice were sacrificed at day 7. As previously reported (23), the results of lung viral titer assessment were significantly different between variants (Fig. S6B), with WA1 having the highest titer and Omicron the lowest titer. The SARS-CoV-2 mRNA transcripts for E and N genes were detectable using nucleic acid amplification within small intestinal tissue (Fig. S6C and D) with trends mirroring those observed for viral plaque assays from the lung (Fig. S6B). To evaluate the gut microbiota, we performed 16S-Seq on 55 samples, resulting in 435,539±7,099 (mean±sem) high-quality reads/sample (Table S2B).

**Fig 3:**
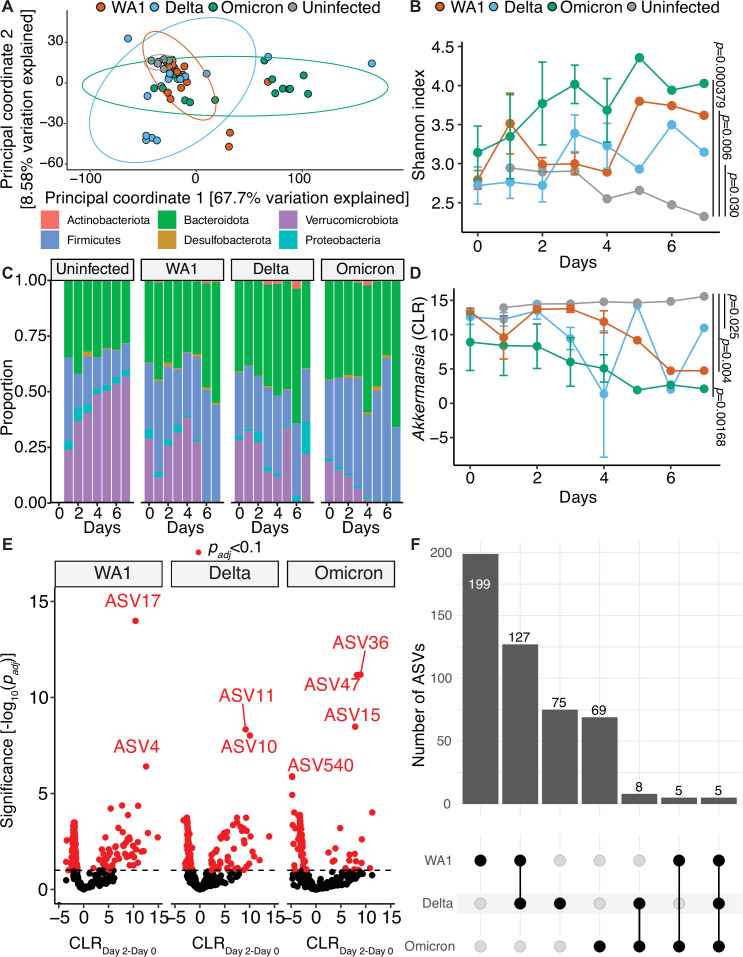
Severe acute respiratory syndrome coronavirus 2 (SARS-CoV-2) variants have significant and heterogeneous impact on the mouse gut microbiota. (**A**) Principal coordinate analysis of 16S-Seq data for mouse samples from all groups and timepoints. Ellipses represent 95% confidence intervals assuming a multivariate t-distribution. (**B**) Shannon index for 16S-Seq data colored by variant of concern against the days after infection on the x-axis (Days). (**C**) Phylum-level taxonomic summaries for each group plotted against days after infection (Days). (**D**) *Akkermansia* Centered Log Ratio (CLR) for each variant by day post infection. (**B, D**) P values displayed are results of two-way analysis of variance and reflect values comparing each variant to the uninfected group. (**E-F**) A linear mixed effect model comparing differential abundance of variants across all timepoints for each each variant was created and in (**E**) a Volcano plot of p_adj_ versus difference in CLR between day 2 and day 0 abundance displayed and in (**F**) features with an p_adj_ < 0.1 and similar effect between variants are shown. Features with the lowest p_adj_ are annotated in (**E**). *n* = 10 unique cages with 55 total samples from 50 mice in (**A–D**) and *n* = 9 unique cages and 18 unique samples in (**E–F**).

All SARS-CoV-2 variants led to a dramatic and significant impact on the mouse gut microbiota. Visualization of all three variants compared to the uninfected mice showed clearly distinct patterns of grouping (Fig. 3A, WA1 *r*^*2*^ = 0.247, *P* = 0.00530; Delta *r*^*2*^ = 0.135, *P* = 0.0499; Omicron *r*^*2*^ = 0.395, *P* = 0.000900); however, they did not reach statistical significance by PERMANOVA testing after adjusting for longitudinal sampling (*P*_*adj*_ = 1, all variants). PERMANOVA testing on a per variant basis comparing changes with day post infection revealed trends to changes over time that did not reach statistical significance when adjusted for cage (WA1 *r*^*2*^ = 0.187, *P* = 0.0254, *P*_*adj*_ = 0.0882; Delta *r*^*2*^ = 0.169, *P* = 0.0663, *P*_*adj*_ = 0.215; Omicron *r*^*2*^ = 0.169, *P* = 0.0883, *P*_*adj*_ = 0.467). Bacterial diversity increased over time following infection with all three variants (Fig. 3B, Shannon index, WA1 *P* = 0.006, WA1:Time interaction *P* = 0.031, Delta *P* = 0.030, Delta:Time interaction *P* = 0.008, Omicron *P* = 0.000379, Omicron:Time *P* = 0.049). A linear mixed effect model confirmed that Shannon index changed by time on a per variant basis (WA1 *P* = 1.45e-6; Delta *P* = 3.15e-11; Omicron *P* = 2.19e-6). We observed differences in relative abundance at the phylum level for four phyla between groups (two-way ANOVA for *Verrucomicrobiota*, *P* = 6.09e-8; *Firmicutes*, *P* = 1.29e-5; *Bacteroidota*, *P* = 9.08e-4; and *Proteobacteria*, *P* = 1.10e-2). There was a striking loss of *Verrucomicrobiota* in Omicron-infected mice and a subtle reduction in *Proteobacteria* in WA1-infected mice, with reciprocal shifts between *Firmicutes* and *Bacteroidota* in these groups (Fig. 3C). Notably, the relative abundance of *Akkermansia* diminished over time in response to infection with WA1, Delta, and Omicron (Fig. 3D).

Given the multi-strain design of our experiment, we sought to understand whether the impact of SARS-CoV-2 on the gut microbiota was variant-specific. The baseline gut microbiota was indistinguishable between groups (PERMANOVA *r*^*2*^ = 0.260, *P* = 0.754, *P*_*adj*_ = 1) with zero significantly different ASVs (*P*_*adj*_ > 0.1) between each variant and uninfected controls. We leveraged the within-subjects design of this longitudinal dataset, comparing ASV relative abundance overtime for each variant including their baseline samples using a linear mixed effect model (Fig. 3E). Most differentially abundant ASVs were variant-specific (Fig. 3F). The number of significant ASVs matched SARS-CoV-2 viral load, with each successive variant leading to a less pronounced microbiota shift: WA1 (336 ASVs), Delta (215 ASVs), Omicron (87 ASVs) (Table S3A; Fig. S6B). Shared responses were highest for WA1 and Delta (127 ASVs) with five ASVs consistently altered in all three variant groups (Fig. 3E and F). Taken together, these results indicate that the impact of SARS-CoV-2 on the gut microbiota varies between viral strains and has decreased over time.

**Fig 4:**
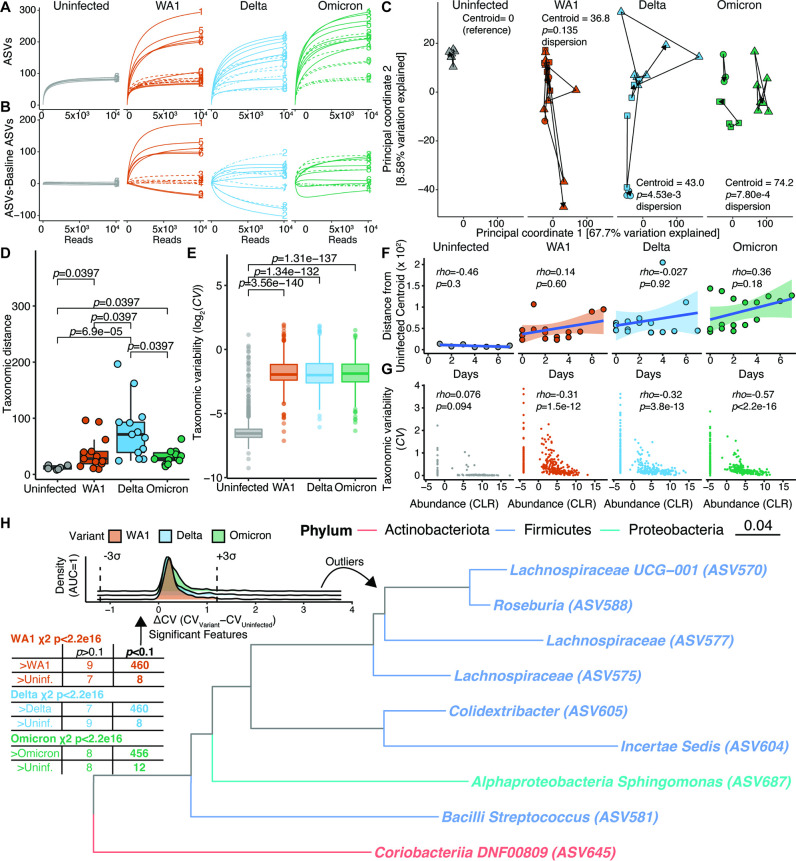
Severe acute respiratory syndrome coronavirus 2 (SARS-CoV-2) destabilizes the mouse gut microbiota. (**A**) Rarefaction curves for uninfected and WA1-, Delta-, or Omicron-infected mice. (**B**) Rarefaction curves of subsequent timepoints normalized to the baseline timepoint for each longitudinally sampled group. (**A-B**) Each longitudinally sampled group is delineated by a different line type (solid, dashed, dot-dashed for mice sampled to days 7, 4, and 2, respectively). The day post infection is annotated. (**C**) Principal coordinates 1 and 2 of 16S-Seq data separated by group to facilitate visualization. The distance from the uninfected reference group’s centroid is displayed for the right three plots (p values, β-dispersion comparing each variant to the uninfected reference group with Tukey’s multiple comparisons adjustment). Each shape represents a longitudinally sampled cage. (**D**) Subsequent distances between successive points in (**C**) for a given cage are plotted and grouped by SARS-CoV-2 variant. (**E**) Coefficients of variance (CV) for each 16S amplicon sequence variant (ASV) is plotted by SARS-CoV-2 variant. (**D–E**) Dunn’s post-hoc test of Kruskal-Wallis analysis of variance. (**F**) Euclidean distance from the centroid of the uninfected group is plotted by day of sampling after infection. A Spearman’s correlation coefficient and p value are annotated. A line of best fit with 95% confidence interval is superimposed. (**G**) A correlation between Taxonomic variability and Abundance is displayed, and a Spearman’s correlation coefficient is annotated. (**H**) An *F* statistic was used to compare ASV variability between each SARS-CoV-2 variant and the uninfected group (p_adj_ shown). A two-by-two table is shown of the findings with a χ2 test annotated below the table for each variant. Δ*CV* for ASVs with a significant test for any Variant are plotted as a density curve, with three standard deviations above and below 0 annotated. The features corresponding to outlier ASVs are displayed in the phylogenetic tree. All displayed features had higher CV values in all three Variants compared to the uninfected group. *n* = 10 unique cages with 55 total samples from 50 mice.

Finally, we tested if SARS-CoV-2 infection of the K18-hACE2 model would recapitulate the microbiota destabilization phenotype that we observed in humans. We generated rarefaction curves from the longitudinally sampled mice in a given cage. Rarefaction curves were stable over time in uninfected controls, but showed extensive heterogeneity in all three SARS-CoV-2-infected groups (Fig. 4A). The range of ASVs detected was 84–95 (controls), 70–320 (WA1), 53–249 (Delta), and 84–324 (Omicron). We generated rarefaction curves normalized to the baseline timepoint of longitudinally sampled cages and observed a similar pattern of heterogeneity and absence of an obvious temporal association (Fig. 4B). Microbiota instability was also clear by PCoA (Fig. 4C), with a significant increase in β-dispersion for Delta and Omicron. Changes in the gut microbial variation (Fig. 4D) and species variability (Fig. 4E) were significantly increased in infected mice. Notably, microbiota shifts were not significantly associated with time post infection (Fig. 4F), consistent with PERMANOVA testing (WA1 Time *r*^*2*^ = 0.0919, *P* = 0.0862; Delta Time *r*^*2*^ = 0.0717, *P* = 0.213; Omicron Time *r*^*2*^ = 0.0649, *P* = 0.145).

**Fig 5:**
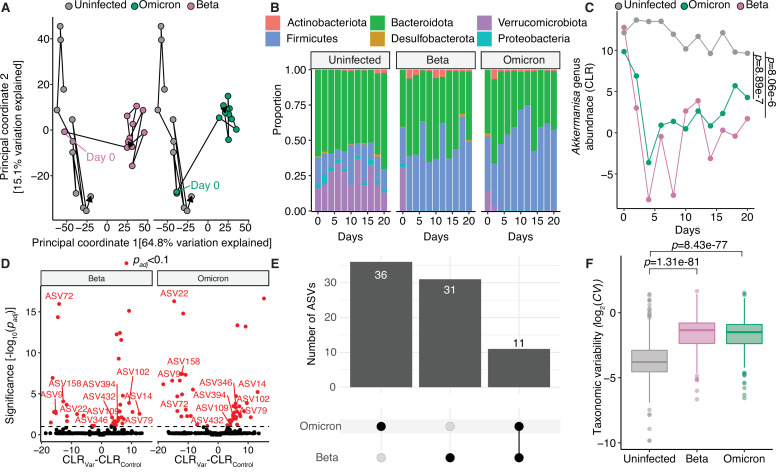
Mild severe acute respiratory syndrome coronavirus 2 (SARS-CoV-2) infection in wild-type mice result in destabilization to the gut microbiota and loss of *Akkermansia*. (**A**) Principal coordinate analysis of C57BL/6J mice comparing communities after Beta and Omicron variant infection are shown relative to uninfected mice. (**B**) Phylum-level taxonomic summaries for uninfected or Beta- or Omicron-infected mice sampled from days 0 to 20 after inoculation. (**C**) *Akkermansia* genus, Centered Log Ratio (CLR) for each variant plotted against day of sampling post-inoculation. P values, two-way analysis of variance comparing each variant to the uninfected group. (**D–E**) A linear mixed effects model was created for each variant. (**D**) Volcano plot of p_adj_ versus difference in CLR abundance. (**E**) Differentially abundant amplicon sequence variants (ASVs) shared/distinct between variants. Overlapping ASVs in (**E**) are annotated in (**D**). (**F**) Taxonomic variability is plotted comparing uninfected and Beta- and Omicron-infected mice. *n* = 15 mice with five mice per group in three cages, and 33 total longitudinally collected samples.

Next, we sought to identify which bacterial taxa were most variable following SARS-CoV-2 infection in mice. As expected, there was a negative correlation between CV and ASV abundance (Fig. 4G). In uninfected mice, the correlation between CV and abundance was nearly bimodal with features having a CLR value <0 having high CV values compared to those with mean CLR values above 0. In contrast, during infection, there was significantly increased CV across organisms of all abundances, though the negative correlation between CV and ASV abundance was still preserved (Fig. 4G). We utilized *F* tests to compare ASV variability between each variant and the uninfected controls, revealing that nearly all ASVs were more variable in infected mice (Fig. 4H, χ^2^ table). We then generated a distribution of CV between variants and the uninfected group that reflected these shifts and selected outliers that were three standard deviations above or below an equal CV value between groups. The outliers among this group were exclusively more variable in the infected mice and primarily from the *Firmicutes* phylum (Fig. 4H, density curves and phylogenetic tree). Taken together, these findings support a causal and strain-specific role of SARS-CoV-2 in microbiota instability.

### SARS-CoV-2 impacts the gut microbiota in the absence of lung pathology

Given the robust shift in the gut microbiota in response to Omicron, the mildest variant in the K18-hACE2 model, we hypothesized that the immunological or other types of host responses to viral inoculation could alter the gut microbiota independent of host disease. To test this hypothesis, we turned to wild-type C57BL/6J mice that are resistant to severe lung pathology from SARS-CoV-2 infection (24). We infected C57BL/6J mice with Beta and Omicron variants and longitudinally collected stool samples for 20 days (Fig. S6E). We performed 16S-Seq on 33 samples, resulting in 398,412±10,679 (mean±sem) high-quality reads/sample (Table S2C). Live virus was detectable in the lungs at day 2 post infection; however, we did not detect any live virus in the gastrointestinal tract (Fig. S6F).

Remarkably, Beta and Omicron variants resulted in a pronounced shift in the gut microbiota of C57BL/6J infected mice by day 2 post infection (Fig. 5A, Beta *r*^*2*^ = 0.622, *P* = 0.000100; Omicron *r*^*2*^ = 0.590, *P* = 0.000100). The adjusted PERMANOVA was not significant, likely due to insufficient power (Beta *P*_*adj*_ = 1, Omicron *P*_*adj*_ = 1). Beta-infected mice showed greater bacterial diversity compared to uninfected mice, although Omicron-infected mice C57BL/6J mice did not show this trend (Fig. S7A). Phylum-level abundance was significantly altered in response to infection (two-way ANOVA for *Verrucomicrobiota*, *P* = 6.97e-8; *Firmicutes*, *P* = 1.54e-8; *Bacteroidota*, *P* = 1.10e-2; and *Proteobacteria*, *P* = 1.07e-6). *Proteobacteria* were depleted in Beta- and Omicron-infected mice, though not as marked or pronounced as the depletion of *Verrucomicrobiota* in these same groups (Fig. 5B). Correspondingly, *Akkermansia* were depleted in both Beta- and Omicro-infected groups (Fig. 5C). We confirmed reduction in *Akkermansia* was most significant immediately after infection in the wild-type mice infected with either Beta or Omicron (Beta and Omicron combined linear mixed effect model *P* = 0.00522 for days 0 through 4 post infection; linear mixed effect model *P* = 0.383 including all timepoints). No ASVs were significantly different between the uninfected and Beta- and Omicron-infected mice at baseline (*P*_*adj*_ > 0.1). Numerous ASVs were identified as being depleted or elevated by both Beta and Omicron, with 11 ASVs overlapping between both groups (Fig. 5D and E; Table S3B). Finally, while there was no difference in β-dispersion or distance traveled between sampling points for Beta- or Omicron-infected mice compared to the uninfected group (Fig. S7B and C), the variability of taxonomic features was significantly greater in mice infected with Beta or Omicron (Fig. 5F).

## DISCUSSION

Our results in humans and mice demonstrate that the gut microbiota is destabilized following mild cases of SARS-CoV-2 infection; however, the cellular and molecular mechanisms responsible remain to be elucidated. SARS-CoV-2 most likely impacts the gut microbiome through effects on host immune or epithelial cells (28). However, despite its minimal impact on the host, Omicron still led to a dramatic collapse of the mucin-dependent gut Verrucomicrobium *A. muciniphila*. These results may suggest that SARS-CoV-2 can lead to dysfunction of the intestinal goblet cells, due to either direct infection or the increased level of intestinal cytokines. While early work on WA1 suggested that it cannot bind to goblet cells (29), this work conflicts with recent data demonstrating goblet cell hyperplasia in response to severe SARS-CoV-2 infection (28). The impact of Omicron on *A. muciniphila* was notable and suggests the continued evolution of SARS-CoV-2 may have preserved its ability to perturb the intestinal mucosa.

The downstream consequences of microbiota instability for COVID-19 pathophysiology will be important to assess. Persistent symptoms have been observed in a subset of adults characterized principally by fatigue, headache, anosmia, and dyspnea though impacting every organ system (16). In our own study, the majority of Cases met WHO definitions of SARS-CoV-2-related symptoms persisting >90 days from diagnosis (Table S1). One Case subject did not participate in follow-up symptom reporting. A recent report showed findings similar to ours in that taxonomic changes in the gut microbiome were captured as late as 6 months after SARS-CoV-2 infection and were linked to prolonged symptoms, though instability of the gut microbiome was not assessed (30). In our own cohort, symptoms related to SARS-CoV-2 included cold symptoms, anosmia, and gastrointestinal symptoms (Table S1). Gastrointestinal symptoms such as nausea, abdominal pain, or diarrhea may be exacerbated by more recently evolved variants either through their direct impact on the intestine and/or resulting destabilization of the gut microbiome caused by infection. We observed a trend to greater dispersion between subjects with long-term symptoms compared to those without (dispersion *P* = 0.318 Cases with or without long COVID symptoms), though were underpowered to compare these groups.

It is also important to consider the impact of disruptions in the gut microbiota to host responses in other contexts. SARS-CoV-2 significantly alters the pulmonary immune response (23). *Akkermansia muciniphila* has established links to promoting activation of exhausted *T* cells (31), and eliciting high-quantity mucosal IgA responses (32). Disrupting *A. muciniphila* or other bacterial species that control immune function could be important as the host balances viral clearance with tissue damage that might lead to outcomes like acute lung injury or prolonged symptoms after infection (33).

Importantly, we discovered a marked instability in the human gut microbiota following SARS-CoV-2 infection, complicating efforts to identify bacterial genes, pathways, and taxonomic groups that consistently differentiated Cases and Controls in humans. Across all data types and groups in our study, instability was the hallmark of SARS-CoV-2 infection. This data is in line with a concept that has been referred to as the “Anna Karenina Principle,” wherein disease-associated microbial communities are distinct from the microbiotas of healthy individuals but lack shared features (34). Similar observations have been made in the context of broad-spectrum antibiotics, autoimmunity, and enteric bacterial infections (34); however, this is to our knowledge the first evidence that mild cases of a respiratory virus infection can lead to microbial community-wide instability in the gastrointestinal tract. Longitudinal studies of other common viral infections in the outpatient setting will be important in order to test the generalizability of these findings, coupled to larger cohorts of SARS-CoV-2 patients. Furthermore, the individualized nature of the microbiota’s response to infection highlights the potential for incorporating microbiome signatures into precision medicine.

Our study has several limitations. Although the CHIRP samples analyzed in this study are a vanishingly rare commodity of SARS-CoV-2-naïve individuals following initial infection, the number of subjects was small and each individual was unevenly sampled. In addition, we had limited metadata regarding infection-associated changes in diet or other lifestyle factors that could compound microbiome susceptibility, including the social distancing practices of each participant. Furthermore, we may have been insufficiently powered in our human study to see consistent effects of SARS-CoV-2 on the gut microbiota. The duration and number of independent cages/experiments of our mouse studies were limited due to the logistical challenges of performing experiments under ABSL3 conditions.

Despite these limitations, our data clearly show that the gut microbiota can be destabilized, at least in some individuals, following mild SARS-CoV-2 infection. These changes were independent of disease severity and distinct between SARS-CoV-2 variants, potentially due to variant-specific effects on goblet cell function and mucosal integrity. It is notable that SARS-CoV-2 has reached virtually every corner of the world (35). While trends toward milder infections are a cause for celebration, it will be important to consider that mild infections have the potential for months-long destabilization of the gut microbiota, and that more recently evolved variants still have a pronounced impact on the gut microbiota in mouse models. These results will be important to revisit and evaluate in the future as we approach the next era of the pandemic and move toward understanding the long-term impact of prior SARS-CoV-2 infection for our health and the health of our microbial co-conspirators.

## MATERIALS AND METHODS

### CHIRP subject enrollment

Subjects were recruited to the CHIRP study and provided informed consent (IRB 20-30588). Subjects were asked to produce a nasal PCR indicating SARS-CoV-2 status at the time of enrollment. Subjects underwent survey-based questionnaires at visits and provided stool samples which were frozen at −80°C. One participant (CHIRP-4108) had symptoms but did not have a positive PCR test at the time of study enrollment, with subsequent research-based PCR testing as inconclusive. CHIRP-4108 is the child of CHIRP-4107 and CHIRP-4109. Two subjects, CHIRP-4100 and CHIRP 4106 (a Case and Control from separate households) reported antibiotic exposure prior to study enrollment. For the purposes of this study, only subjects with both a definitive positive PCR swab and symptoms are referred to as Cases, and all other individuals are Controls. Household relationships are listed in Table S1.

### Mouse SARS-CoV-2 infection models

Protocols for animal use were approved (AN169239-01C) by the Institutional Animal Care and Use committees at the University of California, San Francisco and Gladstone Institutes. Mice were housed in a temperature controlled-specific pathogen-free facility with a 12-h light–dark cycle and *ad libitum* access to water and laboratory rodent chow. The study involved intranasal infection of 10^4^ plaque-forming units (PFU) of 6–8-week-old female K18-hACE2 mice with Delta, Omicron, and WA1 variants of SARS-CoV-2. In the case of C57BL/6J mice, the mice were infected with 10^3^ PFU of Beta or Omicron variants of SARS-CoV-2. All stool samples from a cage of mice were pooled into one tube with DNA/RNA shield 1–5 cages of infected mice sampled per timepoint. Where possible, two of the aggregated samples were selected for sequencing; otherwise, the resulting slurry was sequenced. In the case of K18-hACE2 mice, a full cage was euthanized at 2, 4, and 7 days post infection for lung viral titer assessment or if meeting criteria for sacrifice on days 5 and 6. The lung and gut tissues were analyzed for viral titer using plaque assays as described (23), with lung tissue data published previously.

### Stool DNA extraction

Stool DNA was extracted using a modified protocol within a BSL2 biological safety cabinet given subjects had known infection with SARS-CoV-2. Samples were handled on dry ice, and containers were decontaminated before and after use with 70% ethanol. Briefly, samples were extracted using phenol–chloroform and 5% hexadecyltrimethylammonium bromide. Samples underwent two rounds of bead-beating for 45 s at a rate of 5.5 m/s, and underwent heat denaturation for 15 min at 65℃. Polyethylene glycol was used to precipitate DNA, which was then washed in 70% ethanol. This method has been described previously (36). For mouse stool samples, roughly two stool pellets were selected from the DNA/RNA shield mixture and processed for 16S-Seq as described below.

### 16S rRNA gene amplicon sequencing

DNA was amplified using Kapa-HiFi Hotstart (KK2502, Kapa Biosystems) using primers to 16S-V4 regions (V4-515F - TCGTCGGCAGCGTCAGATGTGTATAAGAGACAGGTGCCAGCMGCCGCGGTAA, V4-806R - GTCTCGTGGGCTCGGAGATGTGTATAAGAGACAGGGACTACHVGGGTWTCTAAT) on a BioRad CFX 384 real-time PCR instrument with four serial 10-fold dilutions of extracted DNA template. Individual sample dilutions in the exponential phase were selected using an OpenTrons OT2 for subsequent indexing PCR using a dual GoLay error-correcting index primers (37). DNA concentration was measured using a PicoGreen assay (P7589, Life Technologies, South San Francisco, CA, USA), and samples were pooled at equimolar concentrations. Agencourt AMPure XP magnetic beads were used to purify the pooled PCR product, and the samples were subsequently sequencing on an Illumina MiSeq using 15% PhiX spiked in for sequencing. Mouse samples were amplified using V4 primers as previously described (38). All sequencing was paired, with human 16S as 270 bp and mouse 16S as 150 bp fragments. Amplicon reactions were pooled at equimolar concentrations and purified using the Agencourt AMPure XP magnetic beads. The pooled library was loaded onto the Illumina NextSeq 550 platform using 40% PhiX spiked in for sequencing.

### 16S rRNA gene sequencing analysis

Primer sequences and adatpers were trimmed using the cutadapt plugin in QIIME2 prior to analysis. DNA sequences underwent quality filtering, denoising, and chimera filtering utilizing DADA2 (39) as implemented in QIIME2 (40). Taxonomy was assigned to amplicon sequence variants (ASVs) using the SILVA v138 database (41). Two negative control samples were processed in this manner. Greater than 98% of both negative controls reflected chimeric reads and were ~10^2^ lower in final read content than the lowest positive control. Negative control samples were subsequently removed from analysis. ASVs were filtered for those found with at least 10 reads in three samples and subsampled to even sampling depth using MicrobeR (42).

Samples underwent a PhILR transformation using the *de novo* generated phylogeny made using the PhILR package (43). β-Dispersion was assessed in this distance matrix using the function beta.disper in the R package vegan with resulting *p* values were adjusted using Tukey’s multiple comparison test, and permutational multivariate analysis of variance (PERMANOVA) testing was conducted using the adonis2 function with the following formula (distance matrix (DM) ~ Household + COVID_Status + Days After PCR test) (25). For PERMANOVA testing, the adonis2 command from the vegan package (25) was used, and blocks of permutation were set to 10,000 and restricted by individual subject using the how function in base R using participant ID. For unadjusted PERMANOVA testing, 10,000 unrestricted permutational blocks were used. For Fig. 2, distances for individuals from which successive measurements were available were selected from the larger PhILR distance matrix, and initial measurements (e.g., 0 distance) were excluded. A linear mixed effect model with the following formula [Centered Log Ratio (CLR) ~ COVID_Status + (1|Subject ID)] was iterated over all ASVs using lmerTest for Fig. 1, a model fitting CLR ~ Day_Post_Infection|Cage was fit including all sampled timepoints for Fig. 3 and Table S3A, and a model fitting fitting CLR ~ Variant + Day_Post_Infection + (1|Cage) was fit for Fig. 5 and Table S3B (44). ASVs for whom the model failed due to singularity were excluded from the analysis. Resulting p values were corrected using the p.adjust command in R and the Benjamini-Hochberg correction. The mean difference between abundance of day 2 and day 0 samples was chosen to plot across the x-axis in Fig. 3, given this maximized the number of independent replicates based on our experimental design. Calculation of coefficients of variance (CV) was completed by transforming CLR or normalized metagenomic data to ranks, and then dividing the mean of a feature by its standard deviation using the group by command and on a per sample basis iterated across all features for 16S-Seq, taxonomic MGS, and functional MGS data. For 16S-Seq data, F-statistics were obtained by comparing ranks between Cases and Controls for all ASVs using the var.test command in R, and adjusting resulting p values with a Benjamini-Hochberg correction. A *ΔCV* was calculated with negative values being greater in Controls and positive values being greater in Cases. A density curve was plotted with *ΔCV* values, and values demarcating three standard deviations outside of a *ΔCV* of 0 of this curve are displayed in Fig. 2F. The outliers of this distribution are displayed in the phylogenetic tree.

An analysis of variance (ANOVA) for baseline samples from the mouse experiments with the following formula was iterated over all ASVs (CLR ~ Variant). For wild-type mice, this was done comparing uninfected mice to both Omicron and Beta-infected mice. For mouse 16S-Seq data, baseline timepoints were excluded from PERMANOVA testing. PERMANOVA testing was restricted by longitudinally sampled cages by using the how function in base R and 10,000 permutations. For unadjusted PERMANOVA testing, 10,000 unrestricted permutational blocks were used with the following formula (DM ~ Variant + Days Post Infection). Baseline samples from all K18-hACE2 infected mice were compared using PERMANOVA testing with 10,000 permutations and adjusted for cage using the how function in base R with the following formula (DM ~ Variant). Differences in *Akkermansia* were determined by comparing all ASVs assigned to the genus *Akkermansia* between groups after CLR transformation of genus ASVs. Two-way ANOVA was calculated using the anova_test tool in the rstatix package, where the indicated dependent variable in the figure was a function of two independent variables (e.g., SARS-CoV-2 variant and days post infection).

### Metagenomic sequencing

Extracted DNA was prepared using a Nextera XT DNA library preparation kit from Illumina, and methods were followed as specified by the manufacturer. Eighteen out of 58 samples lacked sufficient concentration to create 50 ng of sample material, and for these, 20–30 μL of samples were used to provide the maximum amount of template material at tagmentation and PCR. Samples were amplified using six cycles of PCR using the IDT Illumina Type B indexing primers. Sample DNA concentrations were estimated using a PicoGreen assay (P7589, Life Technologies), pooled to equimolar concentrations using an OpenTrons OT2, and cleaned using the Agentcourt AMPure XP magnetic beads. The resulting library was sequenced on an Illumina NovaSeq instrument at UCSF’s Center for Advanced Technology using paired end sequencing with 150 bp fragments.

### Metagenomic analyses

Shotgun libraries were processed using Humann3 with unstratified pathway data or gene families as outputs (45). Normalized abundance was calculated as Abundance normalized by Genome Equivalents as estimated by MicrobeCensus (46). Histograms of feature counts by number of samples present were created, and a cutoff of feature presence in three samples was used for pathway data or gene family data. While all gene family data trimmed in this manner was used to construct distance matrices and related analyses, low abundant features were trimmed and only the top 20,191 gene families were evaluated for associations with SARS-CoV-2 using a linear mixed effect model and represented in [Fig F2] Fig. 1E. Pathway and gene family data were filtered to reads per kilobase per million*10^3^ cutoffs of greater than 0.0005. A Canberra distance matrix was calculated for filtered functional data with subsequent PERMANOVA testing utilizing adonis2, and nMDS plots were created using the metaMDS command (25). PERMANOVA testing, dispersion assessment, and distances between points were done as described for 16S-Seq.

### SARS-CoV-2 E and N protein quantitative PCR

Mice were infected with the indicated variants of SARS-CoV-2, and RNA was extracted from small intestinal tissue that underwent homogenization. Quantitative polymerase chain reaction (qPCR) was conducted using SYBR Green. qPCR was conducted for SARS-CoV-2 envelope (E) and nucleocapsid (N) genes (E gene forward primer: 5′-ACAGGTACGTTAATAGTTAATAGCGT-3′; reverse primer: 5′-ATATTGCAGCAGTACGCACACA-3′, N gene forward primer: 5′-AAATTTTGGGGACCAGGAAC-3′; reverse primer: 5′-TGGCACCTGTGTAGGTCAAC-3′) and normalized to Gapdh gene (forward primer: 5′-AGGTCGGTGTGAACGGATTTG*-*3′; reverse primer: 5′-TGTAGACCATGTAGTTGAGGTCA-3′). Reactions were 10 µL and conducted in 384-well plates using an annealing temperature of 60℃ on a CFX 384 Touch Real-Time PCR Detection System (Bio-Rad).

### Social distancing variable measurements

We obtained population-level social distancing variables from Cubiq. Cubiq provided two pieces of data for each zip code and date combination. The Cubiq Mobility Index (CMI) quantifies movement in users in a given region per day. Movement was calculated using a derivative factor indicating the distance between opposite corners within a box around locations for users on a given day. CMI values can be interpreted as follows: 5–100,000 m; 4–10,000 m; 3–1,000 m; 2–100 m; 1–10 meters. The “Home Percentage” variable is the estimated number of users sheltering-in-place, which is calculated by the number of users moving less than 330 ft from home on a given day.

### Statistical analysis

Data were analyzed either in this R version 4.0.4, or an enterprise version of R studio 3.5.1 for 16S-Seq data and R version 3.6.1 for shotgun data on the Wynton Computing cluster (a co-op-based computing cluster at UCSF). Unless otherwise stated, data were analyzed using the following software packages in R: tidyverse, ggplot2, and rstatix (47, 48).

## Data Availability

All sequencing data pertaining to this manuscript has been uploaded to the NIH Sequence Read Archive under the accession number PRJNA885137 (MGS) and PRJNA907010 (16S-Seq). MGS data were depleted of human reads prior to upload. Metadata for each sample is included in the SRA deposited samples and corresponds to identified sample names within the supplemental tables of this manuscript.

## SUPPLEMENTAL MATERIAL

Fig S1 mbio.00889-23-s0001.pdf Clinical study sampling scheme.

Fig S2 mbio.00889-23-s0002.pdf Lack of a consistent signature of mild SARS-CoV-2 infection based on 16S-seq and metagenomic data.

Fig S3 mbio.00889-23-s0003.pdf Feature variability negatively correlates with abundance in Cases and Controls.

Fig S4 mbio.00889-23-s0004.pdf The human gut microbiome is less stable following mild SARS-CoV-2 infection as measured by 16S-Seq and metagenomic data.

Fig S5 mbio.00889-23-s0005.pdf Variation in population-level social distancing practices.

Fig S6 mbio.00889-23-s0006.pdf The ABSL3 SARS-CoV-2 infection model and associated viral titers.

Fig S7 mbio.00889-23-s0007.pdf Minimal changes to gut microbial diversity or markers of dispersion in C57BL/6J-infected mice.

TABLE S1 to S3 mbio.00889-23-s0008.xlsx Supplemental tables for patient metadata and sequencing results.

ASM does not own the copyrights to Supplemental Material that may be linked to, or accessed through, an article. The authors have granted ASM a non-exclusive, world-wide license to publish the Supplemental Material files. Please contact the corresponding author directly for reuse.
