# Supplementary material for: Mild SARS-CoV-2 infection results in long-lasting microbiota instability
Source: mBio. 2023 Jun 9;14(4):e00889-23. doi: 10.1128/mbio.00889-23 (PMC10470529; doi:10.1128/mbio.00889-23)
Supplement: Fig S1 — Clinical study sampling scheme. [file mbio.00889-23-s0001.pdf]

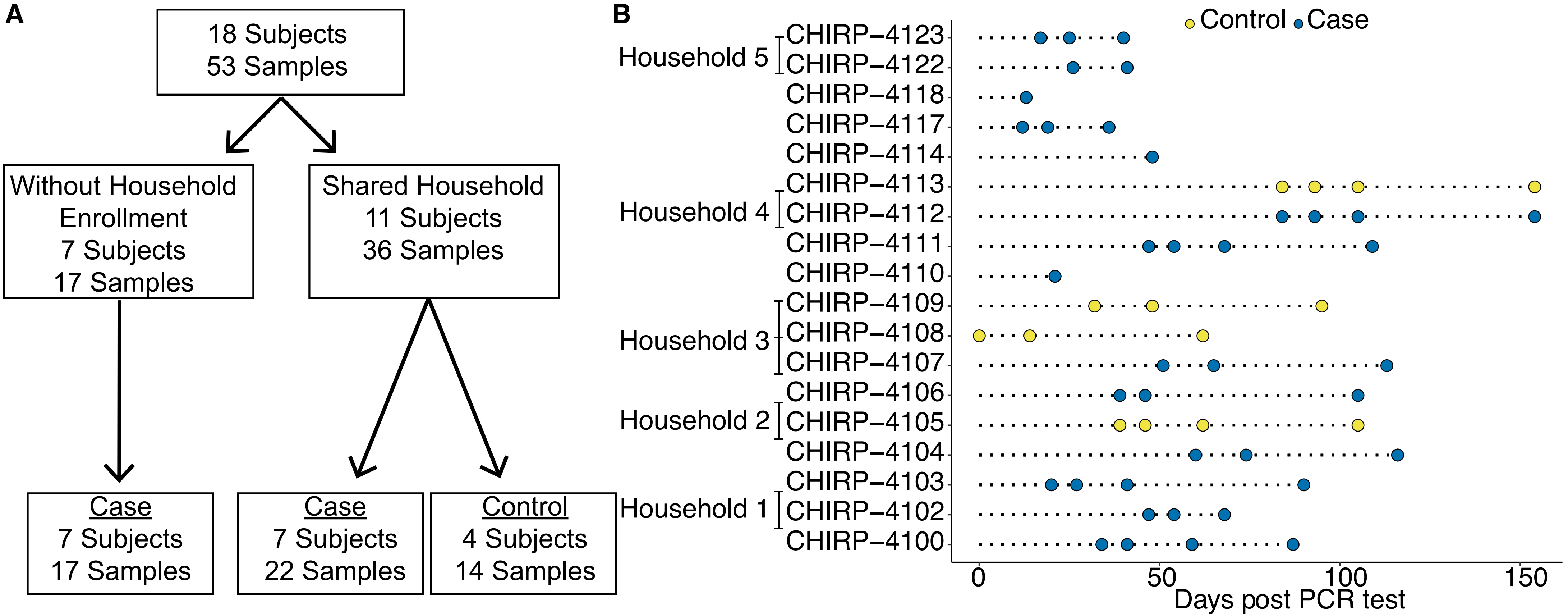

FIG S1 Clinical study sampling scheme. (A) CONSORT diagram for the overall CHIRP study and the microbiome sub-analysis. **(B)** Sample collection with respect to positive test result for SARS-CoV-2 subject in household. Brackets indicate individuals from the same household as described in **Table S1**.
