# Supplementary material for: Mild SARS-CoV-2 infection results in long-lasting microbiota instability
Source: mBio. 2023 Jun 9;14(4):e00889-23. doi: 10.1128/mbio.00889-23 (PMC10470529; doi:10.1128/mbio.00889-23)
Supplement: Fig S2 — Lack of a consistent signature of mild SARS-CoV-2 infection based on 16S-seq and metagenomic data. [file mbio.00889-23-s0002.pdf]

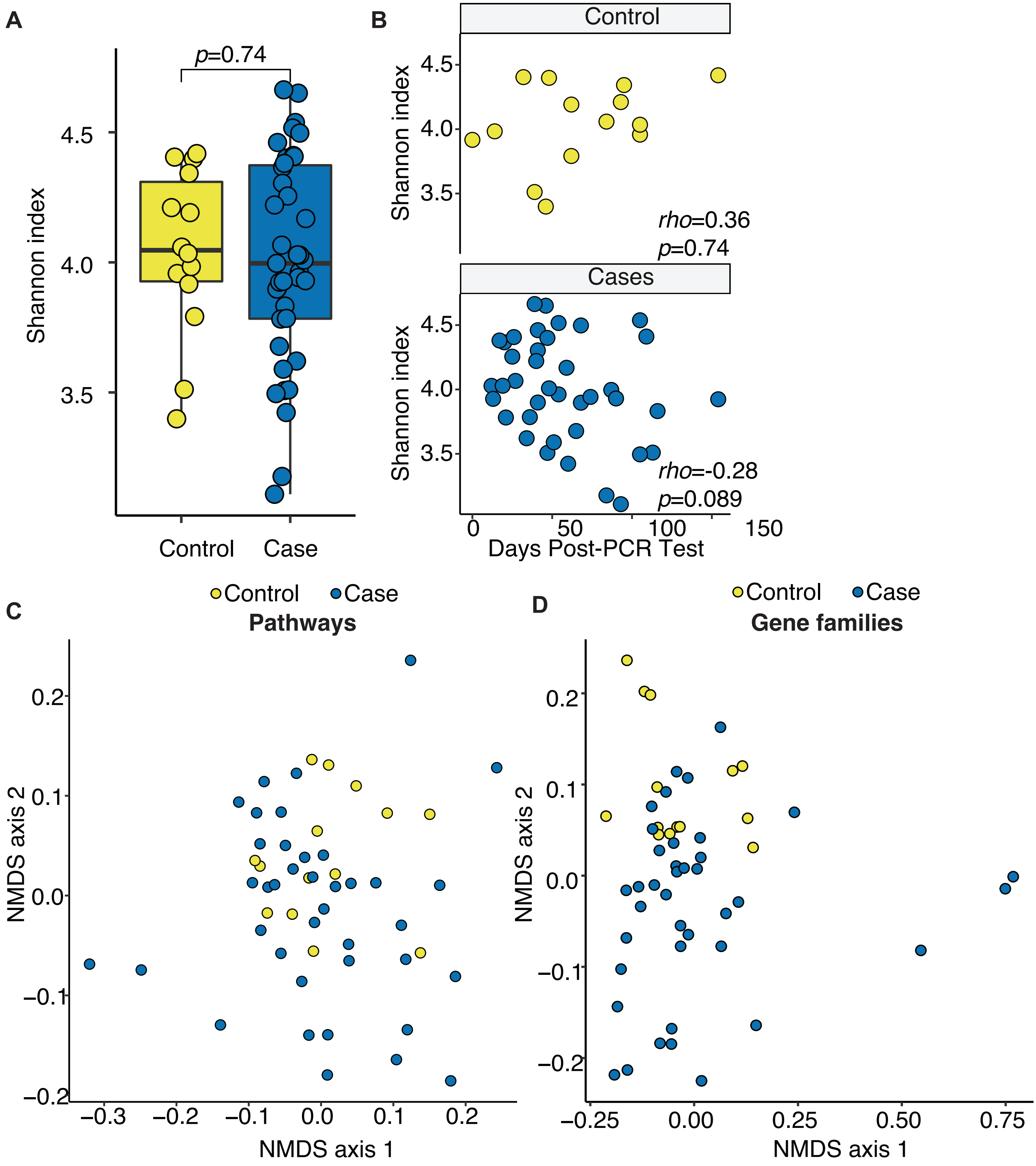


**FIG S2 Lack of a consistent signature of mild SARS-CoV-2 infection based on 16S-seq and metagenomic data. (A-B)** 16S-Seq data. **(C-D)** MGS data. **(A)** Microbial diversity was comparable between groups based on the Shannon Index (*p*-value, Student's *t* test) and **(B)** was not significantly associated with time post-PCR test (Spearman correlations shown). **(C-D)** MGS unstratified pathway data **(C)** and gene families **(D)** were not significantly different between Cases and Controls (PERMANOVA $p_{adj}$=1, both panels, n=18 subjects, 53 samples).
