# Supplementary material for: Mild SARS-CoV-2 infection results in long-lasting microbiota instability
Source: mBio. 2023 Jun 9;14(4):e00889-23. doi: 10.1128/mbio.00889-23 (PMC10470529; doi:10.1128/mbio.00889-23)
Supplement: Fig S3 — Feature variability negatively correlates with abundance in Cases and Controls. [file mbio.00889-23-s0003.pdf]

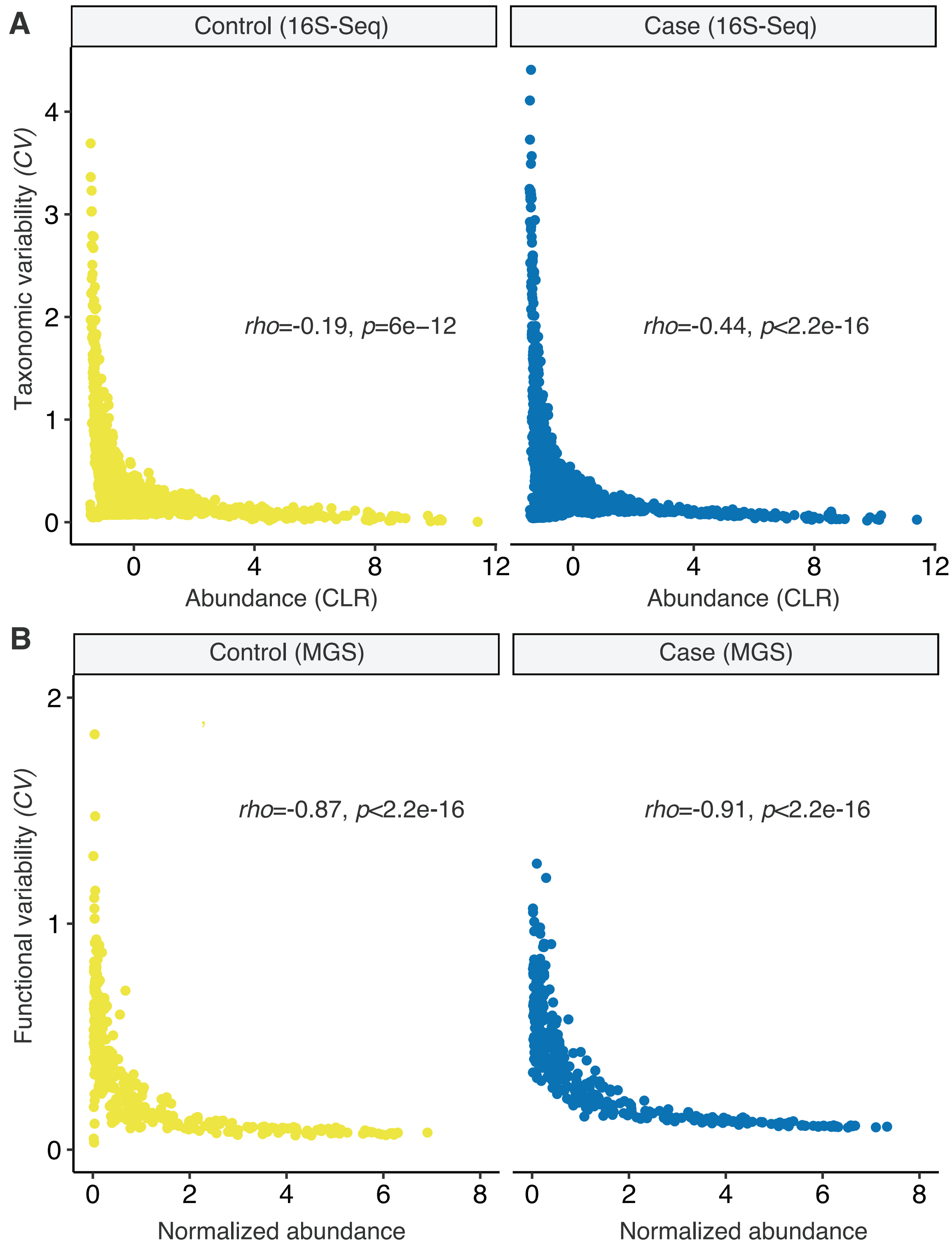

FIG S3 Feature variability negatively correlates with abundance in Cases and Controls. The *CV* of 16S-Seq ASVs **(A)** or MGS Pathways **(B)** was plotted against the abundance of each and separated between Cases and Controls. A Spearman's correlation coefficient is annotated indicating a negative correlation between CV and feature abundance in all groups. n=18 subjects, 53 samples.
