# Supplementary material for: Mild SARS-CoV-2 infection results in long-lasting microbiota instability
Source: mBio. 2023 Jun 9;14(4):e00889-23. doi: 10.1128/mbio.00889-23 (PMC10470529; doi:10.1128/mbio.00889-23)
Supplement: Fig S4 — The human gut microbiome is less stable following mild SARS-CoV-2 infection as measured by 16S-Seq and metagenomic data. [file mbio.00889-23-s0004.pdf]

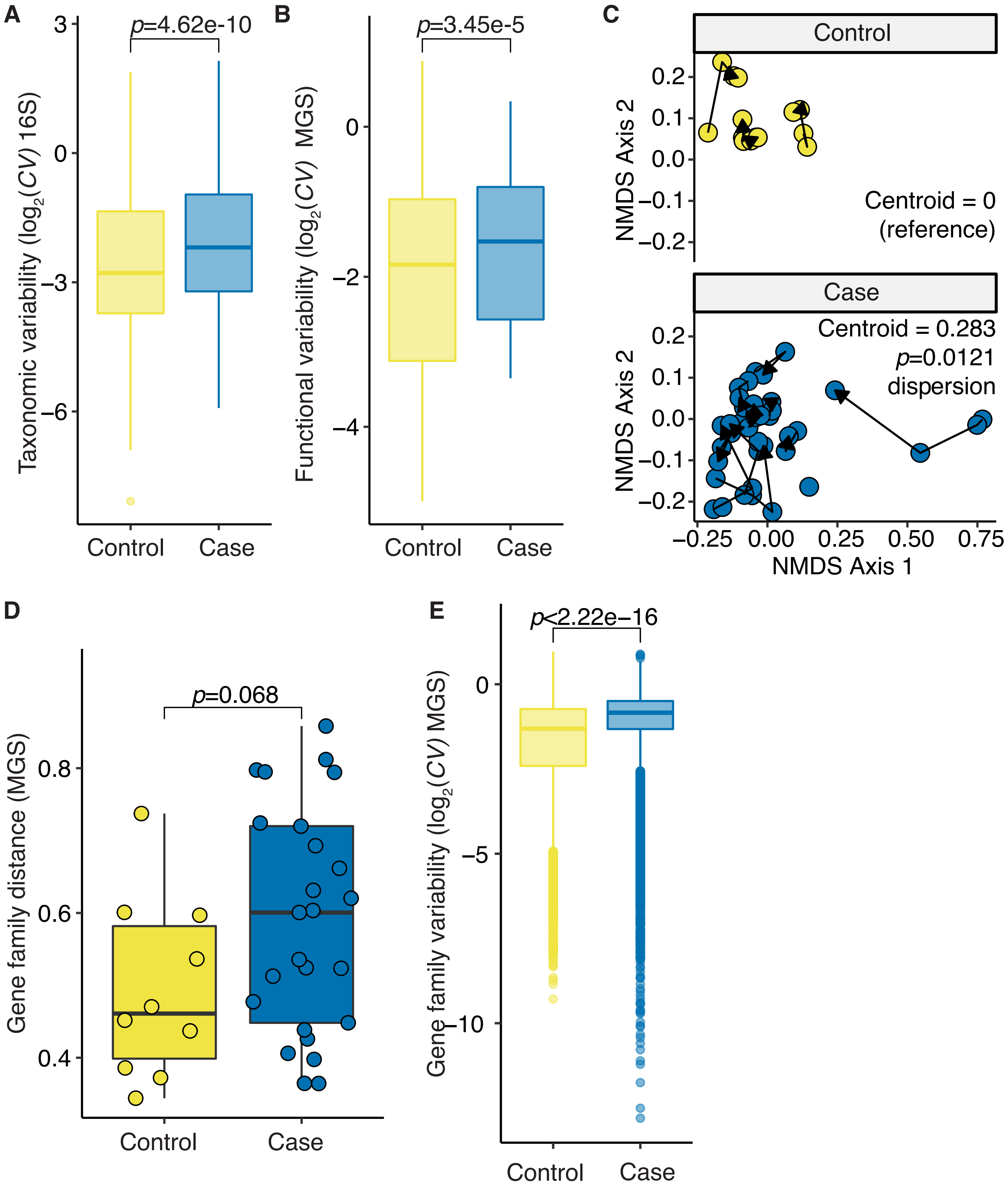

FIG S4 The human gut microbiome is less stable following mild SARS-CoV-2 infection as measured by 16S-Seq and metagenomic data. (A-B) *CV* was calculated for each feature making up **(A)** 16S-Seq and **(B)** Pathway Data. A Dunn's post test of Kruskal-Wallis ANOVA is annotated. **(C)** Principal coordinate analysis of MGS data annotated to gene families. A given subject is connected by a line with arrows indicating successive sample collection; *p*-value for β-dispersion from the vegan package for SARS-CoV-2 positivity is displayed and is calculated between Cases and Controls and adjusted using Tukey's multiple comparisons. **(D)** Subsequent distances between successive points as shown in **Fig. S4A**. Student's *t*-test between groups is annotated. **(E)** *CV* was calculated for each feature making up gene family MGS data. A Dunn's post test of Kruskal-Wallis ANOVA is annotated. n=18 subjects, 53 samples.
