# Supplementary material for: Mild SARS-CoV-2 infection results in long-lasting microbiota instability
Source: mBio. 2023 Jun 9;14(4):e00889-23. doi: 10.1128/mbio.00889-23 (PMC10470529; doi:10.1128/mbio.00889-23)
Supplement: Fig S5 — Variation in population-level social distancing practices. [file mbio.00889-23-s0005.pdf]

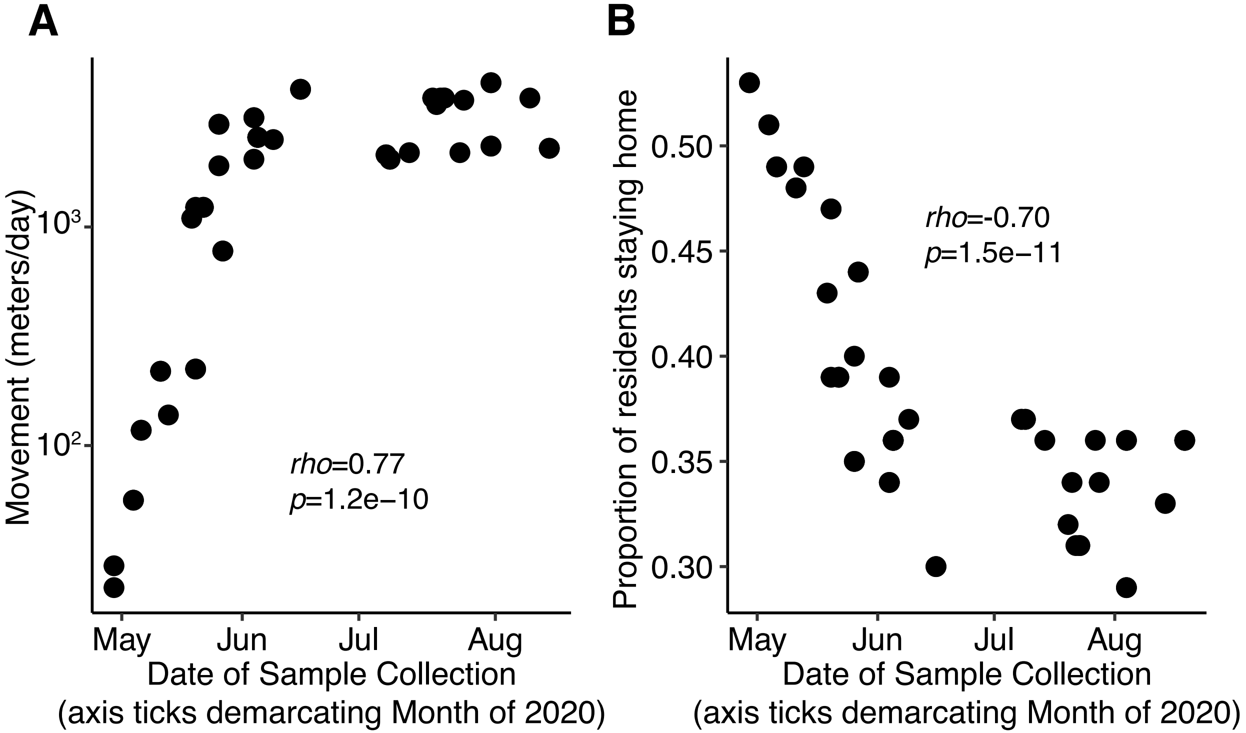

FIG S5 Variation in population level social distancing practices. Population level social distancing data was obtained from the Cubiq organization which utilizes cellular telephone movement of users to approximate movement and number of residents remaining at home. This was done for each sample based on the zipcode of the CHIRP participant and the date of sample collection. **(A)** The Cubiq Mobility Index is plotted on the y-axis and approximately reflects movement in 10^n meters/day where n is Cubiq Mobility Index **(B)** The proportion of residents estimated to reside at home per day by Cubiq is plotted on the y-axis. In both **(A)** and **(B)** the values from Cubiq are plotted against the date of sample collection on the x-axis. Major ticks indicate the month of 2020. A Spearman's correlation coefficient and *p*-value are annotated. n=16 subjects, 48 samples for which this type of data was available from Cubiq.
