# Supplementary material for: Mild SARS-CoV-2 infection results in long-lasting microbiota instability
Source: mBio. 2023 Jun 9;14(4):e00889-23. doi: 10.1128/mbio.00889-23 (PMC10470529; doi:10.1128/mbio.00889-23)
Supplement: Fig S6 — The ABSL3 SARS-CoV-2 infection model and associated viral titers. [file mbio.00889-23-s0006.pdf]

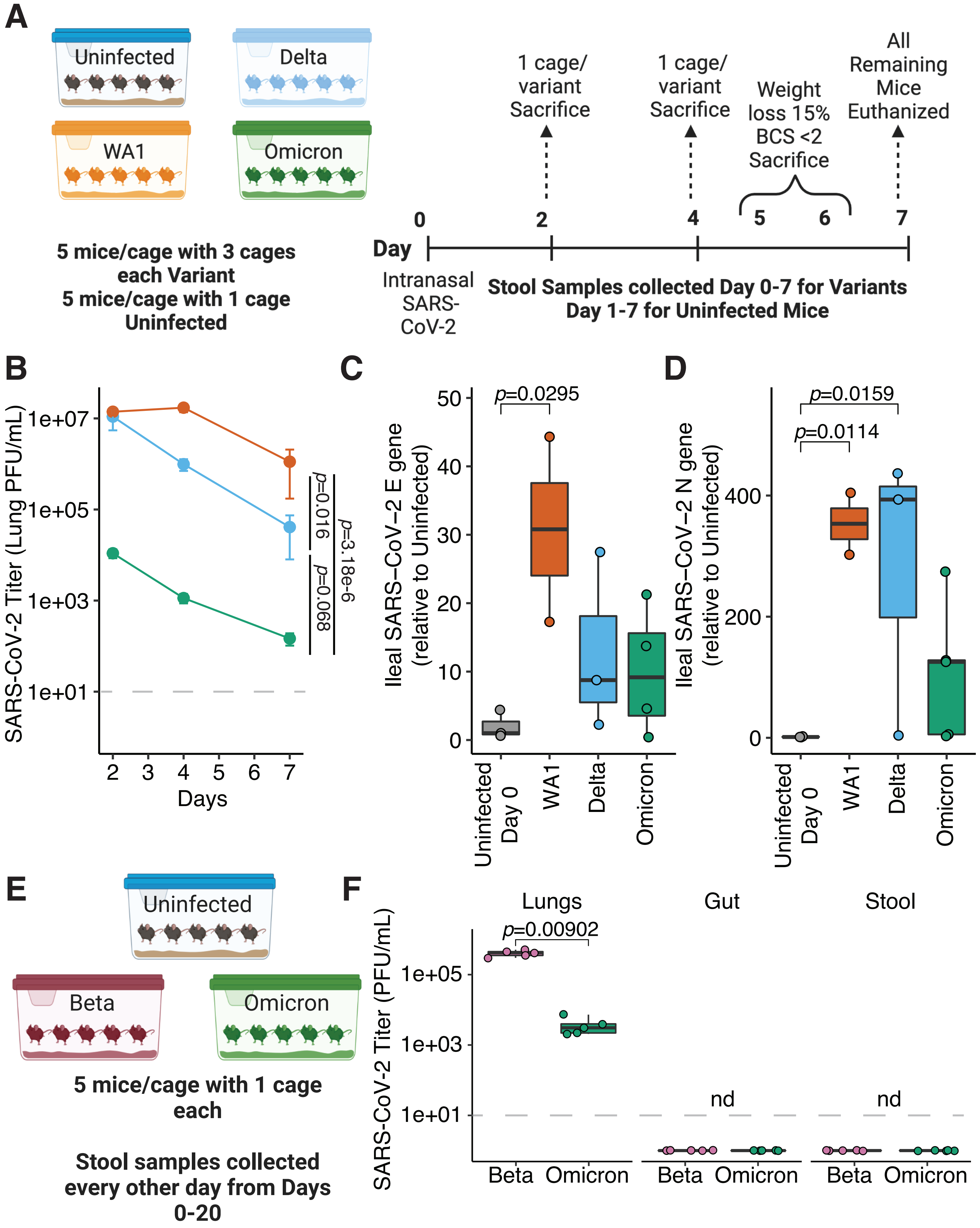

FIG S6 The ABSL3 SARS-CoV-2 infection model and associated viral titers. (A) 50 female mice were housed at a ratio of 5 mice/cage in an ABSL3 facility. Three cages of mice were infected with each variant (n=45 mice, n=15 per variant) and one cage of mice was uninfected (n=5 mice). One cage of mice was sacrificed for each variant at days 2, 4, and 7 for lung viral titer assessment. Any mice reaching a sacrificial endpoint (weight loss of 15% or body conditioning scale (BCS) < 2) were euthanized and removed from the experiment on days 5 and 6. All remaining mice were sacrificed on day 7. **(B)** Lung viral titers for each variant are shown as described in (23) with mean±sem plotted at each timepoint. Statistics represent 2 way-ANOVA and where titer is a function of variant and time, and the *p*-value for variant between the indicated comparisons is displayed. The limit of detection (LOD) for viral plaques is indicated by a gray, dashed line (LOD=10 plaque forming units (PFU) per mL). SARS-CoV-2 envelope (E) gene qPCR **(C)** or nucleocapsid (N) gene qPCR **(D)** was conducted on tissue from animals harvested at day 7 post infection. The unadjusted Dunn's post-test of Kruskal Wallis analysis of variance is displayed for the groups with $p<0.05$ and all non-displayed comparisons are not significant. Uninfected mice were sacrificed and included as controls; given they were never infected with SARS-CoV-2 a day of infection of 0 was assigned in the figure. For **(A)** n=10 unique cages with 55 total samples from 50 mice. In **(B)** n=12 mice for WA1, n=13 mice for Delta, and n=15 mice for Omicron infected mice. In **(C-D)** each data point reflects one mouse (n=2-4 mice per group and timepoint). **(E)** Experimental design for C57BL/6J background infected mice. **(F)** PFUs from indicated body sites (n=5 mice per group) and statistics reflect Mann-Whitney U test between groups for lung samples. Live virus was not detected (nd) from Gut and Stool. LOD=10 PFU per mL and is indicated by a gray, dashed line.
