# Supplementary material for: Mild SARS-CoV-2 infection results in long-lasting microbiota instability
Source: mBio. 2023 Jun 9;14(4):e00889-23. doi: 10.1128/mbio.00889-23 (PMC10470529; doi:10.1128/mbio.00889-23)
Supplement: Fig S7 — Minimal changes to gut microbial diversity or markers of dispersion in C57BL/6J-infected mice. [file mbio.00889-23-s0007.pdf]

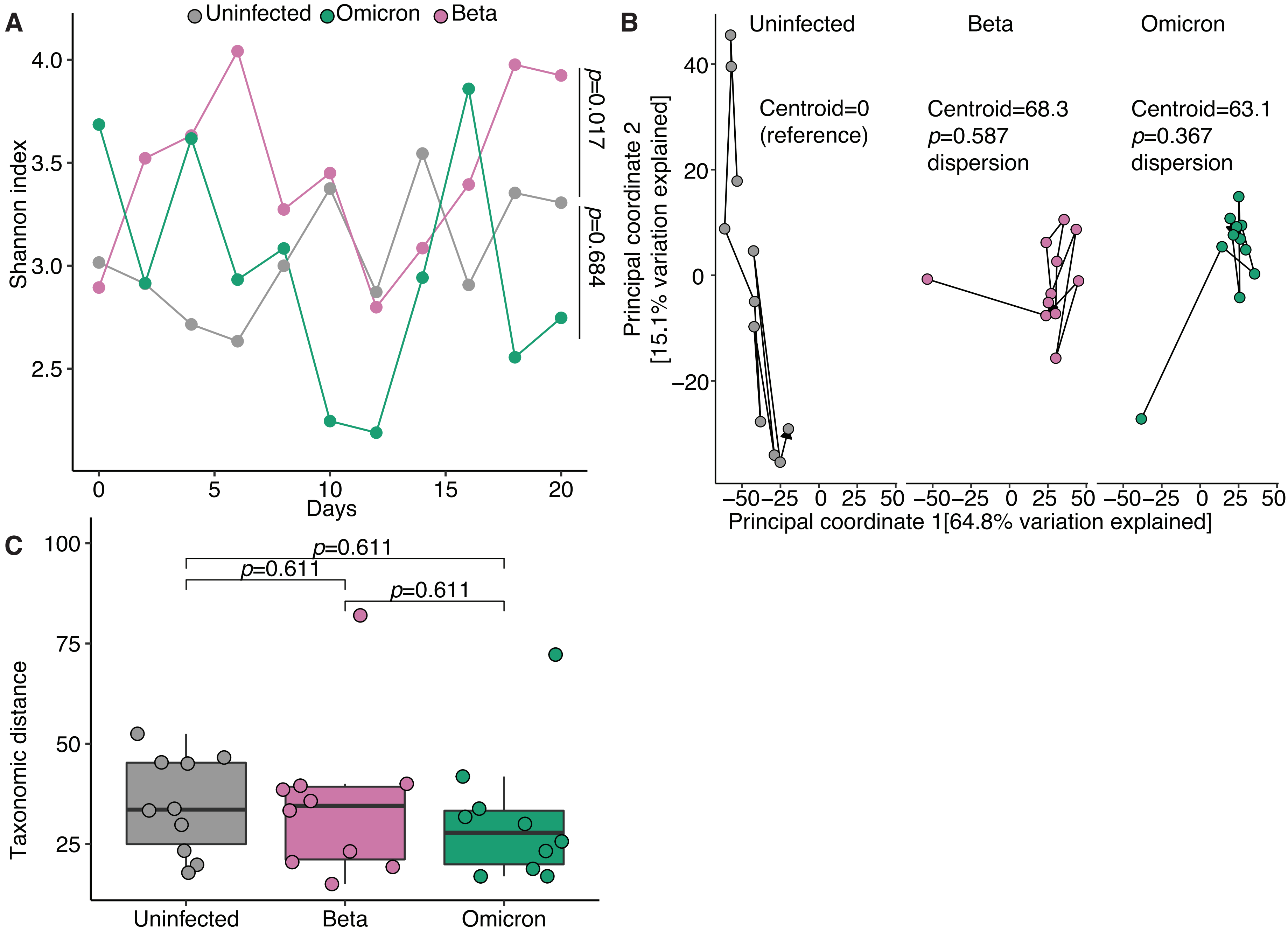

FIG S7 Minimal changes to alpha diversity or markers of dispersion in C57BL/6J infected mice. (A) Alpha diversity is plotted over time with *p*-values annotated showing the statistics for a two way ANOVA comparing the indicated variant to the uninfected group. **(B)** Principal coordinates 1 and 2 of 16S-Seq data for all analyzed samples derived from the same distance matrix and separated by group to facilitate visualization. The distance from the Uninfected reference group's centroid is displayed for the right three panels. The *p*-value for β-dispersion from the vegan package for COVID Status is displayed and compares the variant to the uninfected reference group and adjusted using Tukey's multiple comparisons. **(C)** Subsequent distances between successive points in **(B)** for a given cage are plotted and grouped by variants of concern. Dunn's post-hoc test of Kruskal-Wallis analysis of variance is displayed and adjusted with a Benjamini Hochberg correction. n=15 mice with 5 mice per group in 3 cages and 33 total longitudinally collected samples.
